# Preparation of Eye Instruments before Usage

**Published:** 1913-04-05

**Authors:** 


### PREPARATION OF EYE INSTRUMENTS BEFORE USAGE.

The numerous fine and delicate knives, scissors, probes, needles, and many other instruments used by ophthalmic surgeons for operations upon the eyes require care and attention during the processes of sterilisation greatly in excess of that required for the instruments used in general surgery. The reason of this is that the edge which has to be put on such instruments is far finer, and consequently far more easily damaged and blunted, than in the case of larger and stronger surgical tools. To get round this difficulty various ophthalmic surgeons adopt different devices to secure aseptic instruments which shall still be thoroughly effective for their purpose. But the fact that no one system has attained predominance over all others would seem to show that all have drawbacks as well as advantages.

A recent writer* who has travelled extensively among ophthalmic clinics both in Britain and on the Continent has collected much useful information on this subject. The different methods described and discussed should certainly be thoroughly tested; both surgeons and their theatre sisters may profit by Mr. Thomson's account of his travels and his observations.

It has long been believed, as this author says, that boiling is destructive of the delicate edges and points of sharp-eye tools; the blunt ones, such as retractors, probes, and so forth, are no more likely to be deteriorated by boiling than any other surgical instrument used by the general surgeon. The same belief regarding edged tools is held by many operators who practise general surgery; and it is perhaps exceptional in England to see a scalpel sterilised by boiling in soda solution with the other instruments required for the case. Absolute alcohol is a favourite sterilising medium with many surgeons, and various formalin preparations are used by others. For his own part, Mr. Thomson used to have his knives and needles immersed first in ether, then in alcohol, and then in sterilised water, but of late years he has had them dipped into a steriliser after removal from the alcohol.

On the Continent, on the other hand, most ophthalmic surgeons are boiling their knives for three minutes in the usual 1 per cent. soda solution. Edges and points are protected by special racks in the steriliser. After removal of this rack from the boiling soda the instruments are laid on a sterile towel, where they soon become quite dry, and are then ready for use. Professor Fuchs, for instance, asserts that this treatment has no prejudicial effect upon the blades or edges, provided the steel has been properly tempered. Cataract knives are proverbially apt to lose the razor-edge which is essential to successful operating, but Fuchs has had knives which he has used for forty consecutive operations.

In France dry sterilisation at high temperatures is the vogue. The necessary autoclaves and apparatus are expensive, but the results are said to be very good. One disadvantage which would seem at present to be insuperable is the delay that ensues should re-sterilisation of a contaminated instrument be necessary during the course of an operation, or the sterilisation of one not included in the list of those originally set out. This difficulty practically necessitates the use of an ordinary steriliser as well, in order to avoid vexatious and troublesome delays.

There are still many operators who stick to the chemical methods. Thus Hess uses 5 per cent. carbolic acid, and then dips the instruments into a steriliser. Maddox employs in succession ether, phenol, alcohol, and sterile water. Gunn used a mixture of alcohol and phenol. Others use 5 per cent. phenol only. Herbert has his knives liberally smeared with vaseline and sterilises them by boiling.

Some operators are quite indifferent to the presence of water, saline, or similar bland sterile fluid on their swabs, hands, and instruments. Others prefer everything dry. Mr. Thomson, after a careful comparison of the two methods in various cliniques, pronounces in favour of the dry method, and sketches the ideal procedure thus: The instruments having been boiled in racks in a steriliser, not for too long at a time, the rack or racks are laid on a large sterile towel. An assistant or nurse, wearing sterilised rubber gloves or armed with sterile tongs, lifts the instruments out of the racks and lays them ready on the towel. The surgeon, whose hands are dry, receives the dry instruments from the gloved hands of the instrument assistant or from the tongs with which they have been picked up. It is obvious that this routine of aseptic precaution is applicable to other operations besides those upon the eye, though in them perhaps there is not such a special advantage to be gained by using knives and other sharp instruments dry.

* Ernest Thomson, M.D., *Glasgow Medical Journal*, February 1913, p. 115.

